# Breast Cancer Survivorship Care: Targeting a Colorectal Cancer Education Intervention

**DOI:** 10.3390/jpm5030296

**Published:** 2015-08-06

**Authors:** Sherri G. Homan, Shumei Yun, Bob R. Stewart, Jane M. Armer

**Affiliations:** 1 Public Health Epidemiologist, Missouri Department of Health and Senior Services, Division of Community and Public Health, Office of Epidemiology, 920 Wildwood Drive, Jefferson City, MO 65109, USA; 2 Sinclair School of Nursing, University of Missouri, Columbia, MO 65211, USA; E-Mails: StewartB@missouri.edu (B.R.S.); Armer@missouri.edu (J.M.A.); 3 Chronic Disease and Nutrition Epidemiology Team, Missouri Department of Health and Senior Services, Division of Community and Public Health, Office of Epidemiology, 920 Wildwood Drive, Jefferson City, MO 65109, USA; E-Mail: Shumei.Yun@health.mo.gov; 4 School of Medicine, University of Missouri-Columbia, MO 65212, USA; 5 College of Education, University of Missouri, Columbia, MO 65211, USA

**Keywords:** female, breast neoplasms, colorectal neoplasms, survivors, colonoscopy, focus groups, attitudes

## Abstract

Breast cancer survivors are at risk of developing a second primary cancer. Colorectal cancer (CRC) is one of the leading second primary cancers, and it is often preventable. We developed a multi-component educational tool to inform and encourage women breast cancer survivors to engage in CRC screening. To assess the strengths and weakness of the tool and to improve the relevancy to the target audience, we convened four focus groups of women breast cancer survivors in Missouri. We also assessed the potential impact of the tool on the knowledge, attitudes, and beliefs regarding CRC and collected information on the barriers to CRC screening through pre- and post-focus groups’ questionnaires. A total of 43 women breast cancer survivors participated and provided very valuable suggestions on design and content to update the tool. Through the process and comparing pre- and post-focus group assessments, a significantly higher proportion of breast cancer survivors strongly agreed or agreed that CRC is preventable (78.6% *vs.* 96.9%, *p* = 0.02) and became aware that they were at a slightly increased risk for CRC (18.6% *vs.* 51.7%, *p* = 0.003). The most cited barrier was the complexity of preparation for colonoscopy.

## 1. Introduction

Breast cancer survivors, at almost 3 million, comprise the largest group of cancer survivors recorded in surveillance databases in the United States [1]. There have been great accomplishments in breast cancer survival, with an overall five-year relative survival of almost 90% [1]. If detected at an early, localized stage, the five-year relative survival exceeds 98%. Contributing to these milestones are genetic discoveries [2,3,4], advances in screening [5,6] and personalized therapies [7,8,9,10]. Breast cancer survivors face many of the same health issues as any individual given similar genetic, socio-demographic, and lifestyle characteristics but also have the added health challenges of the potential for recurrence or development of a second primary cancer. Molina-Montes *et al.* conducted a systematic literature review and a separate meta-analysis to evaluate the risk of developing a second primary cancer among female breast cancer survivors. The literature review found statistically significant increased risks in 11 studies ranging from 9% to 40% [11]. The risks varied by the age at breast cancer diagnosis. The meta-analysis that began with 13 studies, excluded one study due to heterogeneity. Thus, combining 12 studies showed a 17% increased risk compared to the general population [11]. In a separate study, Raymond *et al.* found approximately 12.3% of female breast cancer survivors experience a second primary cancer and this risk varied by age (11.7% diagnosed with breast cancer before age 50 and 17% diagnosed at or after age 50) and cancer type [12]. Of the total second primary cancers detected (N = 44,249), breast cancer was the leading second primary cancer (51.8%), followed by colorectal (9.8%) and lung (7.9%) cancers [12]. Other studies also reveal colorectal and lung cancers as substantial proportions of second primary cancers among breast cancer survivors [13,14,15,16].

Colorectal cancer is one of the three most commonly occurring cancers for women (after breast cancer and lung cancer) and men (after prostate cancer and lung cancer) in the United States [17]. It is also the second leading cause of cancer death [17]. Breast cancer survivors have about a 10% or higher risk for developing colon cancer depending on genetic, environmental and other factors including age at the time of the primary breast cancer diagnosis [11,18,19,20]. Some behaviors, such as smoking, are also risk factors for colorectal cancer, and many other types of cancer [21]. While some treatments may also contribute to the etiology of a small proportion of second primary cancers [22,23,24,25], the primary cancer and second cancers often share common risk profiles and suboptimal screening [26,27].

A review of the evidence from well conducted clinical trials and other studies support the effectiveness of the different screening modalities in detecting polyps (*i.e.*, precursor lesions to cancer) and reducing colon cancer incidence and mortality [28]. Although no single screening modality is supported in the guidelines, colonoscopy is the preferred screening tool by many professionals and is used as the standard for comparing other screening modalities. It is associated with a first-exam sensitivity of 90 percent for large polyps and 75 percent for small polyps (<1 cm) [29], lowers the incidence [30,31] and mortality of colon cancer [32,33], and is used for follow-up of positive screening results from other tests. Therefore, colonoscopy is emphasized in the education tool.

In 2012, about two-thirds (65.1%) of the U.S. adult population 50 to 75 years of age met the U.S. Preventive Services Task Force (USPSTF) guideline for colorectal cancer (CRC) screening (*i.e.*, fecal occult blood test [FOBT] within one year, or sigmoidoscopy within five years with FOBT within three years, or colonoscopy within 10 years) with colonoscopy being the most commonly used screening test (61.7%) [27]. In 2013, 58.2% of adults met the USPSTF CRC guideline, which is well below the Healthy People 2020 target of 70.5% [34]. Several studies have found the Health Belief and Transtheoretical Models or their constructs applicable and useful to understanding CRC screening behavior [35,36,37,38,39,40] and inform the framework for this study. While female breast cancer survivors tend to engage in mammography and colonoscopy screening to a greater extent than non-cancer survivors, opportunities still exist to expand screening and promote other preventive practices [41]. In addition, this study is important, as it is the first study to tailor a CRC screening intervention for female breast cancer survivors and builds the foundation for expanding the knowledge base regarding the use of small media to improve the CRC screening rate. Working with a national cancer cooperative group, public health collaborators and health care partners, we developed a multi-component educational intervention for female breast cancer survivors to inform and encourage their engagement in effective screening for CRC [28]. The primary purposes for conducting the focus groups were two-fold: (1) to improve the educational tool and (2) assess its potential to impact participants. This article describes the formative study framework, process and suggestions for improving the relevancy of the CRC educational intervention for female breast cancer survivors. It also describes the potential effects of the tool on the knowledge, attitudes, and beliefs of female breast cancer survivors regarding CRC and screening, particularly colonoscopy.

## 2. Tool Design and Framework

Through the collaborative efforts of a multi-disciplinary group of researchers and scientists and a systematic literature review, the CRC educational intervention concept was formed to focus on female breast cancer survivors. A review for effective strategies to improve CRC screening showed that a physician recommendation is a strong predictor of CRC screening. In addition, when developing a multi-level intervention, including such elements as video, targeted brochure, provider reminder, and small media/decision aids also increase CRC screening [28]. The education tool, the *Power of Prevention*, includes: targeted physician recommendation letter, evidence-based decision aid booklet with stage-of-change assessment, and DVD video. A physician letter was drafted to include CRC burden statistics, the benefits of screening, the problem of the lack of adherence to screening guidelines, and recommendation for colonoscopy. The decision aid booklet presents an overview of colorectal cancer, information on colonoscopy, the various CRC screening modalities, and overall health improvement. It also addresses identified barriers to CRC screening, including lack of knowledge, perception of good health, fear of the test, embarrassment, and unpleasantness of preparing for a colonoscopy. The “*Get Tested for Colon Cancer. Here’s How*” DVD is included with permission from the American Cancer Society (ACS).

There are many psychosocial aspects involved in the potential diagnosis of CRC and screening that vary by individual, can be test-specific, and long-term or transient. This study explored the psychosocial aspects of CRC and screening based on theoretical constructs. Theories help explain the health preventive patterns of people (e.g., why some people engage in screening and others do not) through the psychosocial aspects of attitudes (e.g., fear, embarrassment); beliefs (e.g., perceived health, risks, or benefits); and barriers (e.g., lack of knowledge of CRC and screening, involved preparation for colonoscopy) among other social, demographic, and cost factors [42]. The Health Belief Model [43,44,45,46] and the Transtheoretical (also called the Stages of Change) Model [47] served as the framework for the intervention design and evaluation. The Health Belief model suggests that a person’s belief in a personal threat to health (*i.e.*, susceptibility and severity) together with the perceived benefits of the proposed behavior (pros), barriers (cons), self-efficacy, and cues to action will predict the likelihood of that behavior. The Health Belief Model provided the basis for the risk-benefit assessment. The Transtheoretical Model premise is that people are at different stages of motivational readiness for engaging in health behaviors and this framework serves as the basis for assessing and evaluating the impact of the intervention on increasing readiness to complete a colonoscopy. For this study the definitions of stage of screening are defined as: precontemplation, never had an FOBT/fecal immunochemical test (FIT), sigmoidoscopy/air contrast barium enema (ACBE) or colonoscopy or last FOBT/FIT was more than a year ago, last sigmoidoscopy/ACBE more than five years ago, or last colonoscopy more than 10 years ago; Contemplation, never had or last colonoscopy was more than 10 years ago but giving considerable thought to having a colonoscopy; Action: FOBT/FIT was within the last year, sigmoidoscopy/ACBE within the past five years or colonoscopy was within the past 10 years. While colonoscopy is the primary endpoint for the main study, completion of any CRC-recommended screening modality will contribute to increasing the CRC screening rate and provides the opportunity for early detection.

## 3. Methodology

Five breast cancer survivorship groups were identified via internet searches and referrals from colleagues and the local office of the ACS. All five groups were invited to participate through telephone contact with the group coordinators or presenting the study to the group, and four groups accepted (80%). To target the *Power of Prevention* for breast cancer survivors, a series of four focus groups were held in Missouri, one in each of four cities—Columbia, Jefferson City, Kansas City, and Chesterfield (St. Louis County). The focus group participants were recruited from breast cancer support groups in the four areas and conducted between 13 April and 4 August 2011. A mixed-method design was used to gather information from breast cancer survivors. The focus groups were conducted using a standardized discussion protocol. The discussion protocol included a welcome and introduction with information on the process for the focus group, conveyed the goal of improving the educational tool and assessment questions. The protocol assessed four areas pertaining to the tool: content, packaging, barriers and overall assessment of increased understanding of colorectal cancer screening and motivation to complete colonoscopy. Participants were encouraged to share any other comments or suggestions at the conclusion of the structured questions. The participants were also asked to complete seven-item pre- and post-focus group discussion surveys regarding attitudes, beliefs and practices regarding CRC screening and a 10-point Likert scale on deciding to have a colonoscopy, and a five-item feedback assessment regarding the overall focus group meeting. The survey instruments were comprised of validated questions from previous research on CRC screening [36], behavioral risk factors [48], and expert reviews and input. The draft physician letter and booklet were reviewed and the DVD watched in its entirety. The booklet was reviewed for content, packaging, barriers, improved understanding, and usefulness. The overall usefulness of the focus group discussion was scored as “1” = “not useful”, “2” = “somewhat useful”, “3” = “not particularly useful”, and “4” = “useful”. The questions regarding how understandable was the information was scored as “1” = “not understandable”, “2” = “somewhat difficult”, “3” = “fairly easy”, and “4” = “very easy”. Whether they would recommend the tool to their family or a friend was either “yes”, “no”, or “don’t know/not sure”. Extensive notes and photos were taken during the focus groups by a registered nurse graduate student and public health graphic artist. To further tailor the educational tool, the breast cancer survivors who participated in the focus groups were invited and consented to have their portraits taken by a professional photographer, complete a three-question interview, and provide quotes for encouraging other breast cancer survivors to participate in CRC screening for inclusion in the tool. Incentives for participating in the focus groups included a breast cancer survivorship pen and note pad set ($2.50 each) and framed portraits for women participating in the portrait interviews ($5.00 each).

Data were analyzed using IBM SPSS Statistics v20 (IBM Corporation, Armonk, NY, USA) and Qualitative Data Analysis (QDA) Minor v3.2 (Provalis Research Corporation, Montreal, QC, Canada). Fisher’s Exact test and the Chi Square test were used to compare the changes in knowledge, attitudes, and beliefs before and after the process. The qualitative analysis framework was designed in collaboration with the Alliance oncology nursing committee and included in the protocol. The analysis was conducted with word and themes coded from the meeting notes to align with the protocol categories. The Missouri Department of Health and Senior Services Institutional Review Board reviewed and determined the study to be exempt.

## 4. Results

The focus groups included a total of 43 women breast cancer survivors including 10 (23.3%) African-American women (Table 1). The majority of participants were in the age group 50–64 years (58.1%), had an educational attainment beyond high school (*i.e.*, some college or college graduate) (69.8%), and were covered by health insurance (93.0%). The largest proportion of participants were from Columbia (n = 18, 41.9%) followed by Jefferson City (n = 10, 23.3%), Kansas City (n = 8, 18.6%), and St. Louis (n = 7, 16.3%).

### 4.1. Focus Group Discussions

Overall, all four of the groups liked the multi-component feature of the tool, the color scheme (blue, pink and white space), the pink ribbon logo, the layout, and spiral binding (Table 2). All four groups also agreed that the title could be shortened and suggested various titles could be used in promoting CRC screening; the picture depicting a surgical team with mask should be replaced; and the graphic showing the reach of sigmoidoscopy compared to colonoscopy should be defined, softened and shaded. Three of the groups felt there should be multiple pictures of “*real*” women on the cover; that the language and tone were appropriate, but there were some terms that should be defined. Three groups also stated the length of the booklet was “*just about right*”. One group felt the length was “*a little long*”. However, many participants indicated or provided additional information to be added. The ACS DVD resonated well with the focus groups and three groups suggested connecting the tool to the ACS theme of more “birthdays”. Two of the groups thought the DVD should include younger-looking “*50ish*” people or include people at varying ages.

**Table 1:** Characteristics of female breast cancer survivors participating in the colorectal cancer prevention and screening *Power of Prevention* focus groups, Missouri, 2011.

Characteristic	Breast Cancer Survivors	
	Focus Group Participants	
	Number	Percent
**Overall**	43	100
***Race***		
White, Non-Hispanic	33	76.7
African-American, Non-Hispanic/Hispanic	10	23.3
***Age Group***		
35–49	7	16.3
50–64	25	58.1
≥65	11	25.6
***Education***		
High school	13	30.2
>High school	30	69.8
***Health Insurance Coverage***		
Yes	40	93.0
No	3	7.0

**Table 2:** Focus Groups review of the colorectal cancer prevention and screening *Power of Prevention* education tool with women breast cancer survivors, Missouri, 2011.

Component	Comments	Number of Focus Groups that Referenced Item
		(N = 4)
**Physician’s Letter**	Add opening statement acknowledging breast cancer survivorship, “*what we’ve been through*” and reason for letter	3
	“*Positiveness*”…Connect to ACS theme of more birthdays, birthday cake	3
	Clarify terms—“adults with average risk”; “number of cases per year”; “deaths from invasive colorectal cancer”	3
	Keep the statistics; “*stats make me read further*”, but move effectiveness of CRC screening to beginning	2
	Include alternate options to colonoscopy, if reimbursement issues	2
	Something done “*to care for ourselves*”	1
**Booklet**		
*Content*	Remove scary, surgical picture	4
	Anatomy picture change to a women’s anatomy or sketch, “*perhaps silhouette*”; identify colonoscopy and sigmoidoscopy in picture	4
	More information about preparation; need alternative preparations; repeat that the physician will provide you instructions; like section on preparation tips	3
	Need “*real*” people photos with testimonials; add photo of “*happy couple*” at the end	3
	Add virtual colonoscopy to discussion on tests	2
	Make clear the idea of CRC is preventable and early detection possible; would rather have the information, the “*unknown is more scary*”	1
	Keep statistics on number of cases per year and women and colon cancer	1
	Embarrassment and pain/discomfort section—good	1
	“*Centimeter, may not know or be familiar with size*”	1
	Medications should also include over the counter and herbal remedies to inform the physician	1
	Expand cost section	1
*Packaging*	Shorten title: “*Understanding the Power of Prevention*”; alternate titles “*Power of Prevention*” or “*Power-N-Prevention*”	4
	Color scheme is good, back page could be lighter, “*love pink ribbon*”, layout and like spiral binding;	4
	Liked design of cover, women appeared too young, need more women on cover	3
	Length is good; language and tone appropriate	3
*Barriers*	Preparation, avoid scheduling activities, many trips to the restroom	1
	Discomfort or bad experience may discourage someone from having another colonoscopy	1
	Need someone to relate information given by doctor after the test	1
	Need someone to drive you home	1
**DVD**	It was educational and should be on the Internet; intro could be deleted, talking with doctor seemed unrealistic	3
	Need younger…“*looked older than 50*” and a variety of ages represented in DVD	2
**Pre- and Post-Assessment Surveys**	Further define stage of change decision scale, include thinking about colonoscopy	2
	Need to assess if previously had a colonoscopy and will have another as scheduled	1
**Overall**	Liked combination of tool: letter, book, and DVD	4
	Might not look at DVD after reading the booklet	1

### 4.2. Pre-Post Focus Group Assessment Surveys

All of the female breast cancer survivors (N = 43) returned the pre-assessment questionnaire prior to the focus group; however, due to the short timeframe, fewer women (N = 29) returned the post-assessment questionnaire. Nevertheless, a significantly higher proportion of female breast cancer survivors on the post-assessment strongly agreed or agreed that colon cancer is preventable (78.6% v 96.9%, *p* = 0.02) (Table 3) and became aware that they were at a slightly increased risk for CRC compared to other women their ages (18.6% *vs.* 51.7%, *p* = 0.003). In terms of the health beliefs, perceived susceptibility or perceived risk of developing CRC increased with the majority of breast cancer survivors believing they had a slightly higher chance of getting CRC. There was also a significant increase in knowledge that CRC is preventable. A large proportion of the breast cancer survivors reported having had a colonoscopy in the past 10 years (76.7%), FOBT/FIT in the past year (18.6%), and sigmoidoscopy/ACBE in the past five years (9.3%) and were in the action phase according to the Transtheoretical Model; however, there were some women who had not had any CRC screening (18.6%) and were considered in the precontemplation or contemplation phase of CRC screening. The main reasons cited for wanting to have a colonoscopy or perceived benefits was to find polyps early and to detect colon cancer. The primary reason for not wanting to have a colonoscopy or perceived barriers on both the pre- and post-assessments was the involvedness of the preparation (*i.e.*, diet and cleansing). Not being recommended by a doctor, financial considerations, and concern about discomfort were also cited as barriers. There was an increase in the stage-of-change scale on deciding to have a colonoscopy from a mean pre-score of 8.13 to a mean post-score of 8.52 indicating many were between contemplation and action in deciding to have a colonoscopy, but this change was not significant (*p* = 0.713). The psychosocial aspect of fear declined following the focus group discussion on the post-assessment.

**Table 3:** Attitudes, beliefs and practices regarding colorectal cancer and screening among female breast cancer survivors participating in the colorectal cancer prevention and screening *Power of Prevention* focus groups, Missouri, 2011.

Indicator	Scale	Pre-Assessment		Post-Assessment		*p*-Value
		Number	% ^a^	Number	% ^a,b^	
Colon cancer is preventable	Strongly agree/agree	33	78.6	31	96.9	0.02 *
	Strongly disagree/disagree/don’t know or not sure	9	21.4	1	3.1	
	N =	42	100.0	32	100.0	
	Missing	1	--	11	--	
When compared to other people your age, would you say that your chances of getting colorectal cancer are:	Much lower/a little lower/about average/much higher/don’t know or not sure	35	81.4	14	48.3	0.003 *
	A little higher	8	18.6	15	51.7	
	N =	43	100.0	29	100.0	
	Missing	0	--	14	--	
A colonoscopy is an exam in which a tube is inserted in the rectum to view the colon for signs of cancer or other health problems. Please tell me how important is having a colonoscopy to you	Extremely important	20	46.5	10	35.7	0.536
	Very important	13	30.2	13	46.4	
	Moderately important	7	16.3	5	17.9	
	Slightly important	2	4.7	0	0.0	
	Not important at all	1	2.3	0	0.0	
	N =	43	100.0	28	100.0	
	Missing	0	0.0	15	--	
Have you had a previous colorectal cancer screening test? (Select all that apply) ^c^	FOBT/FIT in past year	8	18.6	--	--	--
	Sigmoidoscopy or ACBE in past 5 years	4	9.3	--	--	
	Colonoscopy in past 10 years	33	76.7	--	--	
	Never had any of the exams	8	18.6	--	--	
	Would rather not answer this question	1	2.3	--	--	
What is the main reason that you would want to have a colonoscopy or a complete colon examination?	To find colon polyps early	17	40.5	11	37.9	0.062
	To find out if I have colon cancer	9	21.4	11	37.9	
	To take control (assume responsibility for) my health	8	19.0	5	17.2	
	Recommendation from the doctor (or other health care provider	7	16.7	0	0.0	
	Symptom check	0	0.0	1	3.4	
	Other	1	2.4	1	3.4	
	N =	42	100.0	29	100.0	
	Missing	1	--	14	--	
What are some of the reasons you would not want to have a colonoscopy (or another colonoscopy)? ^c^	Diet of clear liquids and taking the laxative for preparation	9	21.4	9	26.5	--
	Not recommended by doctor	6	14.3	2	5.9	
	No need/no symptoms	6	14.3	0	0.0	
	Financial reasons, cost, insurance	5	11.9	4	11.8	
	Pain/physical discomfort	4	9.5	4	11.8	
	Too much trouble/can’t get around to it/not enough time	4	9.5	2	5.9	
	Fear or worry about finding cancer	3	7.1	1	2.9	
	Embarrassment	1	2.4	1	2.9	
	At my age, don’t need anymore	1	2.4	2	5.9	
	Lack of transportation	0	0.0	0	0.0	
On the scale, mark where you are in deciding to have a colonoscopy from “0” undecided to “10” have decided to have	0 to < 3	3	8.3	0	0.0	0.713
	3 to < 5	1	2.8	1	4.0	
	5 to < 7	5	13.9	5	20.0	
	7 to < 10	7	19.4	5	20.0	
	10	20	55.6	14	56.0	
	N =	36	100.0	25	100.0	
	Missing	7	--	18	--	

^a^ Missing excluded from calculating percent; ^b^ May not sum to 100 due to rounding; ^c^ Number may exceed total participants or not sum to 100 due to selection of more than one option; * Statistically significant change from pre- to post-assessment; -- not applicable.

### 4.3. Feedback and Portrait Interviews

An overwhelming majority of women (95%, n = 40) responded that the discussion was useful ($\bar{X}$ = 3.9). When asked what was most useful, the women responded: “*Learning about increased risk for colon cancer with breast cancer*”; “*Booklet and DVD compliment*”; “*Missouri is taking action about this issue*”; and “*You are convincing people not to be afraid of a colonoscopy*”. Most women thought the content was “very easy” (70%) or “fairly easy” (27.5%) to understand ($\bar{X}$ = 3.7). The part least helpful was the picture of the doctors with masks (10%). Suggestions included: more information on preparation and alternatives, develop and post information on a website, emphasize periodic exam, and establish partnerships to distribute the information and tool. The majority (87.5%) would recommend the *Power of Prevention* educational tool to their family or a friend. Eleven women consented, scheduled appointments for portraits, completed the brief interview, and provided quotes. One breast cancer survivor stated “*I think colorectal cancer screening is a very good thing and the reason why—I’ve lost two dear friends to colon and breast cancer… so I think it’s very, very important that we get that [screened]*.” Another survivor stated “*I truly believe that early detection will save lives and so it’s important that we get screened for colorectal cancer*.” In the brief interview, a breast cancer survivor summarized:

We’ve learned more over the last years about mammography but I think for a lot of women the breast cancer is just such a shock to the system that we also don’t think about other kinds of cancers that we can get and I think that it’s hard because just because you get one kind of cancer doesn’t mean you aren’t going to get another which is the same reason why I’m always wearing sun screen and protection. You like to think I’ve been through this awful experience with breast cancer it means nothing’s going to happen, but, unfortunately, other kinds of cancers can happen. And again I believe that anything that I can do to prevent another kind of cancer and keep my body as healthy as possible is something that I am interested in for myself and for others. I think we need to really watch how we’re taking care of our bodies so not only do we not want to have a recurrence of breast cancer but we also want to be able to prevent something like colon cancer if there were opportunities.

## 5. Discussion

For individuals at average risk beginning at age 50, the ACS recommends several colorectal cancer screening tests (alone or in combination) and include: FOBT or FIT every year; or stool deoxyribonucleic acid (DNA) test every 3 years; or flexible sigmoidoscopy (FSIG) or double-contrast barium enema (DCBE), or computed tomographic colonography (CTC) every 5 years; or colonoscopy every 10 years [49]. Several national organizations recommend these CRC screening methods [50]. Contrary to this, the USPSTF CRC screening guidelines are currently limited to FOBT (or FIT), sigmoidoscopy or colonoscopy and recommend routine screening only until age 75 [51]. These guidelines are in the process of being updated through a comprehensive, systematic review of all the tests including blood-based biomarkers such as methylated Septin 9 (mSEPT9) DNA assay [52]. The mSEPT9, particularly the second generation test, is showing promise to improve CRC screening rates and long-term adherence [53,54]. The results from the focus groups and assessments show female breast cancer survivors increased their awareness of their higher risk for CRC after going through the process and have engaged in colonoscopy screening to a higher degree than the general population [27,34], yet there are still barriers that exist such as the extensive preparation, lack of a physician’s recommendation, no symptoms and cost that prevent some women from engaging in colonoscopy or other screenings. While this study was limited to breast cancer survivors from one state and small sample size, these preliminary results indicate a potential that the education tool reduces some of the identified barriers and has a positive effect on knowledge, attitudes, and beliefs regarding CRC and screening.

Smoking, excessive alcohol intake, obesity, physical inactivity and not eating healthy also increase the risk for CRC [55,56,57,58,59]. The science continues to evolve regarding which and at what “dose” preventive factors and lifestyle changes may play in reducing the risk of colorectal and other second primary cancers. Nevertheless, female breast cancer survivors demonstrated a high level of interest in prevention and assuming responsibility for their health. Hence, the educational tool is being expanded to include information on healthy eating, weight management, and referrals for smoking cessation and counseling. Health care providers need to be knowledgeable about these risk factors and provide education and support to assist cancer survivors to minimize their risk profiles.

## 6. Conclusions

Overall, the focus group participants were receptive to education on CRC screening and liked the multi-dimensional components of the educational tool. The participants provided invaluable information and suggestions to make the intervention more relevant to breast cancer survivors such as expressing recognition for being a cancer survivor early in the material. Over-arching themes included: (1) the preference for gain-framed messages (*i.e.*, those that stress the benefits of the activity for promoting screening); (2) having a colonoscopy is very or extremely important; and (3) the majority agreed or strongly agreed that colon cancer is preventable. Many breast cancer survivors indicated that they had previously or would engage in CRC screening with increased knowledge and support from their health care provider. The most frequent barrier cited to having a colonoscopy was the preparation required and this suggested the need for alternative methods for preparing for the test or other non-invasive screening tests that meet or exceed the standards set by colonoscopy. Based on the information from these focus groups, the educational tool has been updated and redesigned to increase the relevance to target female breast cancer survivors. While the findings of this study were used to enhance this specific tool, these findings have broader primary and secondary prevention implications and provide insights for future behavioral interventions for this population.
